# JinQi Jiangtang Tablet Regulates Gut Microbiota and Improve Insulin Sensitivity in Type 2 Diabetes Mice

**DOI:** 10.1155/2019/1872134

**Published:** 2019-01-10

**Authors:** Ying Cao, Guowang Yao, Yuanyuan Sheng, Li Yang, Zixuan Wang, Zhen Yang, Pengwei Zhuang, Yanjun Zhang

**Affiliations:** ^1^Chinese Materia Medica College, Tianjin State Key Laboratory of Modern Chinese Medicine, Tianjin University of Traditional Chinese Medicine, Tianjin 300193, China; ^2^Tianjin Hospital of ITCWM Nankai Hospital, Tianjin 300100, China

## Abstract

**Background:**

Gut microbiota modulates the barrier function and host inflammatory state in metabolic disease. JinQi Jiangtang (JQJT) tablets are a traditional Chinese medicine for the treatment of diabetes. However, the low bioavailability of its chemical compositions makes it hard to explain the pharmacological mechanisms.

**Method:**

Diabetic mice were orally treated with JQJT tablets for 5 weeks. Fasting blood glucose and the level of HbA1c were measured, and ITT were conducted to determine the insulin improvement effect of JQJT tablets. The regulation effect on gut microbiota was assessed by 16S rRNA gene sequencing on an Illumina HiSeq platform. The concentration of short-chain fatty acids was measured by HS-GC/MS. D-LA leakage experiment and PAS staining were used to check the function of the gut barrier. The levels of the inflammatory cytokines were determined by using an ELISA kit.

**Results:**

This study showed that JQJT tablets downregulated fasting blood glucose and HbA1c and regulated gut microbiota. JQJT tablet-treated groups exhibited a more sensitive reaction after a small-dose injection of short-acting insulin. T2DM mice treated with JQJT tablets showed a higher abundance of *Akkermansia* spp. and lower abundance of *Desulfovibrio*. JQJT tablets increased the concentration of acetic acid, propionic acid, and butyric acid; in particular, butyric acid was significantly increased with respect to the MOD group. Gut mucosal barrier function experiment showed that the level of D-LA was obviously decreased in JQJT tablet-treated groups compared with the model group and the number of goblet cells was significantly increased by JQJT tablet treatment. JQJT tablets could also reduce the levels of TNF-*α*, IL-6, and MCP-1, which were related to insulin resistance.

**Conclusion:**

We demonstrated that JQJT tablets could improve T2DM insulin resistance, regulating the gut microbiota and promoting the production of SCFAs. The mechanism was related to increasing the gut barrier function and reducing the host inflammatory reaction.

## 1. Introduction

Type 2 diabetes mellitus (T2DM) is reaching global epidemic proportions as a result of factors such as energy-rich food and lack of physical exercise. Insulin resistance and low-grade inflammation are the most important characteristics of T2DM. Low-grade inflammation could lead to the development of metabolic disorders, primarily insulin resistance and type 2 diabetes. Although prior studies have implicated the adipose tissue as being primarily responsible for inflammation [1], the latest discoveries have correlated intestinal flora disturbance and impairments in the mucosal barrier with increased activation of the inflammatory pathways [2]. The disorder of gut microbiota, which could be seen as a source of endotoxins, led to the disruption of the intestinal barrier function [3]. And the gut barrier, which is affected by gut microbiota, is important for metabolic endotoxemia and metabolic disorders [4]. These findings raise the relationship of inflammation, insulin resistance of T2DM, and gut microbiota, and the crosstalk between gut microbes and the host affects the inflammatory status of individuals.

Chronic metabolic diseases such as obesity and diabetes in patients are often accompanied by gut microbial dysbiosis. Accumulating evidence has suggested that the gut microbiota could be involved in the development of insulin resistance. And the latest discoveries have correlated impairments in the intestinal mucosal barrier with the development of insulin resistance. These findings have revealed the relationships between the composition of gut microbiota, host low-grade inflammation, and insulin resistance. Transfer of the gut microbiota from lean individuals to those with obesity improves insulin sensitivity in the recipients [5]. It has been reported that germ-free mice do not become insulin resistant when subjected to a high-fat diet [6]. Growing evidence indicates that pharmacological manipulation of the gut microbiota may be a potential treatment for T2DM; previous studies have reported that metformin or a polyphenol-rich cranberry extract could modulate the gut microbiota by increasing the abundance of *Akkermansia* spp., which might contribute to its antidiabetic effects [7, 8]. These results suggest that modulation of the gut microbiota by increasing *Akkermansia* spp. could have beneficial effects on insulin resistance.

Because of the high prevalence of obesity and unavoidable side effects of the currently available antidiabetic drugs, the development of herbal medicinal products for the treatment of diabetes has become a global interest. JinQi Jiangtang (JQJT) tablets are a China Food and Drug Administration- (CDFA-) listed Chinese medicine (Z10920027) for the treatment of diabetes. The good curative effect of the treatment for type 2 diabetes had been proved after many years of clinical application [9]. However, the antidiabetic mechanisms of JQJT tablets are still unclear. A recent study reported that JQJT tablets could reduce HFD-induced insulin resistance by regulating glucose and lipid metabolism, increasing insulin sensitivity through activating the AMPK signaling pathway, and subsequently improving *β* cell function [10]. The researches of JQJT tablets about the active ingredients [11] and quality stability [12] showed that the major biologically active fractions of JQJT tablets are from three herbal components of *Coptis chinensis* (the total alkaloid fraction of *C. chinensis*), *Astragalus membranaceus* (the saponins, polysaccharides, and astragalosides of *A. membranaceus*), and *Lonicera japonica* (the polyphenolic acid of *L. japonica*). These ingredients effectively regulate blood glucose metabolism and related inflammatory responses [13]. Previous studies have indicated that these effective components (berberine, Astragalus polysaccharide and astragaloside, chlorogenic acid) have effects on the regulation of gut microbiota [14–16] and protection of intestinal barrier function [17–19], since gut microbiota regulation is important to improve the host low-grade inflammation and insulin resistance. Therefore, we evaluated the effects of JQJT tablets on improving insulin resistance by regulating the gut microbiota, increasing the gut barrier function, and reducing the host inflammatory reaction.

## 2. Materials and Methods

### 2.1. Drug and Diet

JinQi Jiangtang tablets (JQJT tablets (Z10920027), approved by CDFA) were obtained from Tianjin Pharmaceutical Group Co. Ltd. Longshunrong Pharmaceutical Factory, China. Streptozotocin (STZ) was purchased from Sigma Corporation. Both the NCD (containing 10% fat by energy) and HFD (containing 60% fat by energy) were purchased from Beijing Huafukang Biotechnology Co. Ltd. (Beijing, China).

### 2.2. Animals and JQJT Tablet Treatment

Adult male C57BL/6J mice (12 weeks old) were obtained from Beijing Vital River Laboratory Animal Technology Co. Ltd. (Beijing, China). Mice were housed in temperature-controlled conditions with a 12 h light/dark cycle (lights on: 8:00 AM). They had access to food and water ad labium. As previously reported, the method of high-fat diet feed plus STZ injection was employed to develop the T2MD model. Mice were randomly divided into normal-chow-diet-fed group (NCD) and high-fat-diet-fed group (HFD). After 3 weeks, the HFD group mice were intraperitoneally given a single injection of STZ (120 mg/kg). The NCD group mice were given an injection of equivalent volume of citrate buffer as the control group (CON). Fasting glucose was measured one week after injection of STZ, and mice with blood glucose value > 11.1 mmol/L were used in the following study. The blood glucose value in diabetic mice continued to rise with HFD for another 3 weeks. Then, mice with HFD were randomly divided into the following groups: model group (MOD), low-dose JQJT tablet group (JQD), and high-dose tablet group (JQG, 2-fold of clinical equivalent dosages). Mice were daily given orally either water or JQJT tablets for 5 weeks.

### 2.3. Insulin Tolerance Test (ITT)

Mice underwent an oral insulin tolerance test (ITT). Mice were deprived of food for 4 h, and the ITT was performed after an intraperitoneal injection of insulin (0.75 UI/kg body weight). Blood glucose concentrations were measured at 0 min and 15, 30, 60, 90, and 120 min after insulin injection.

### 2.4. Inflammatory Factors

Inflammatory factors including TNF-*α*, IL-6, and MCP-1 were analyzed using an ELISA kit.

### 2.5. Histology

The distal ileum samples for histological studies were maintained in 4% formaldehyde solution and then embedded in paraffin; 5 *μ*m sections were stained with periodic acid-Schiff (PAS). The results were expressed as the number of goblet cells per intestinal villus.

### 2.6. Analysis of Gut Microbiota

Gut microbiota was analyzed by sequencing the V4 region of 16S rRNA genes. The feces in each group were collected, the CTAB method was used to sample the genomic DNA, after detecting the purity and concentration of DNA by agarose gel electrophoresis, an adequate amount of samples was added into a centrifuge tube, and then the sample was diluted to 1 ng/mL by sterile water. The diluted genomic DNA was used as a template. A 16S rRNA gene fragment comprising the V4 regions (16S (sense) 5′-GTGCCAGCMGCCGCGGTAA-3′ and (antisense) 5′-GGACTACHVGGGTWTCTAAT-3′) was amplified using an optimized and standardized 16S-amplicon-library preparation. The PCR products were purified using an Agencourt AMPure XP-PCR purification system. Sequencing was performed on Illumina MiSeq platform. Use New England Biolabs company NEB Next ® Ultra ™ DNA Library Prep Kit for Illumina database construction Kit for the construction of Library, to build good Library after Qubit quantitative and Library detection, after qualified, using MiSeq computer sequencing.

### 2.7. Short-Chain Fatty Acid Analysis by HS-GC/MS

#### 2.7.1. Sample Preparation

The fecal samples were homogenized, and 0.1 g of fecal sample was added to the head sample bottle (20 mL) with a screw top and mixed with 1 mL of 6% H_3_PO_4_ by ultrasound for 3 min. The head sample bottle was placed in the automatic headspace sampler.

#### 2.7.2. HS-GC/MS Detection Experiment Parameters

An Agilent Technologies 7890A gas chromatography system coupled to an Agilent Technologies 5975c inert MSD quadrupole mass spectrometer (Agilent Technologies, Germany) and equipped with an Agilent Technologies 7697A Headspace automatic injector (Agilent Technologies, Germany) and an DB-FFAP capillary column (30 m × 0.25 mm i.d., 0.25 *μ*m film thickness, Agilent Technologies, Germany) was used to perform an analysis of SCFAs from fecal samples. The headspace was maintained at 80°C with an incubation time of 30 min. The samples (1 mL) were injected in a splitless mode into the column at a temperature of 80°C. Helium was used as the carrier gas at a constant flow rate of 1 mL/min through the column. The initial oven temperature was 50°C, which was maintained for 1 min and then raised to 200°C at a rate of 10°C/min. The temperatures of the ion source and injector were 250°C. The mass detector system was operated in an electron impact (EI) mode with an ionization energy of 70 eV. The data of ions monitored were collected from *m/z* 33 to 200. A qualitative analysis was performed by the National Institute of Standards and Technology (NIST 11) MS library.

### 2.8. Statistical Analysis

Data were expressed as mean ± SEM. Significance of the differences between the groups of mice was assessed using the Student *t*-test. For experiments comparing multiple groups, the differences were analyzed by one-way analysis of variance followed by Duncan's post hoc test. *P* values < 0.05 were considered significant.

The nearest shrunken centroid (NSC) method was used to detect the bacterial genera that were specifically over- or underrepresented within each category. The amount of shrinkage was chosen to minimize the overall misclassification error. It allowed the identification of bacterial genera whose relative abundance was significantly different between the categories. The analysis was performed using the Prediction Analysis for Microarrays package under R software.

## 3. Results

### 3.1. JQJT Tablets Increase Insulin Sensitivity in Type 2 Diabetes Mice

**Figure 1:**
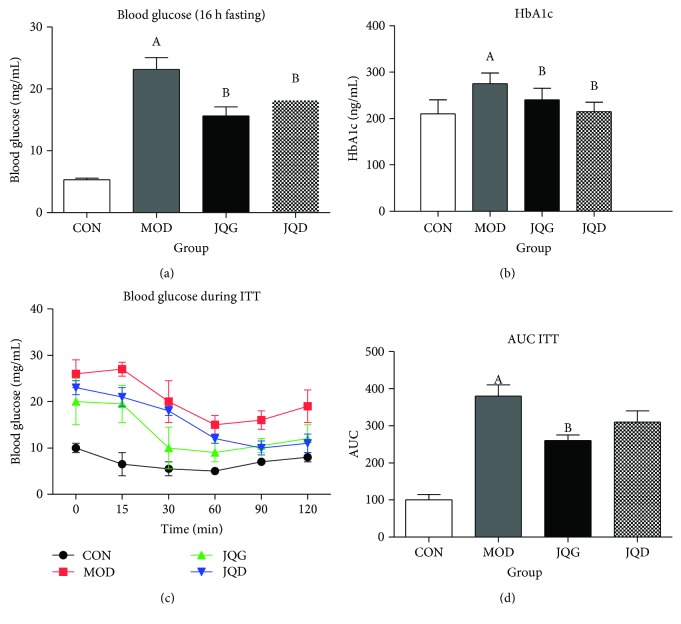
JQJT tablets improve insulin sensitivity in T2DM mice: (a) 16 h fasted for blood glucose, (b) the HbA1c levels, (c) the insulin tolerance test, and (d) the area under the curve (AUC) for the ITT curves (d). Data are expressed as mean ± SEM (*n* = 5 mice/group). Data with different superscript letters are significantly different (*P* < 0.05) by one-way ANOVA.

To investigate whether JQJT tablets reversed insulin resistance in T2DM mice, we administered JQJT tablets to T2DM mice for 5 weeks, fasting blood glucose and serum level of HbA1c were measured, and ITT was performed. We found that 16 h fasting blood glucose (control 5.30 ± 0.29 mg vs. model 23.13 ± 1.93 mg, *n* = 15, *P* < 0.05) and serum level of HbA1c (control 0.21 ± 0.03 mg vs. model 0.28 ± 0.02 mg, *n* = 15, *P* < 0.05) were significantly upregulated in the model group mice. After 5 weeks of JQJT administration, fasting blood glucose (model 23.13 ± 1.93 mg vs. JQG 15.63 ± 1.49 mg and JQD 18.13 ± 1.65 mg, *n* = 15, *P* < 0.05) and HbA1c (model 0.28 ± 0.02 mg vs. JQG 0.24 ± 0.3 mg and JQD 0.22 ± 0.2 mg, *n* = 15, *P* < 0.05) were downregulated significantly (Figures 1(a) and 1(b)). Dietary consumption was also compared between each group; interestingly, we found that JQJT tablets had no effect on dietary consumption (S1).

The response to extrinsic insulin of the mice was investigated after the treatments for 5 weeks by ITT experiments. The results showed that after a small-dose injection of short-acting insulin, the blood glucose levels in the JQJT tablet groups were significantly lower than those in the model mice at each tested time point (*P* < 0.01, Figure 1(c)). The AUC of JQJT tablet-treated groups were significantly reduced (*P* < 0.001) compared with that of model group (Figures 1(c) and 1(d)). Consistent with a previous report [10], these results showed that JQJT tablets could increase insulin sensitivity.

### 3.2. JQJT Tablets Regulate the Gut Microbiota in Type 2 Diabetes Mice

**Figure 2:**
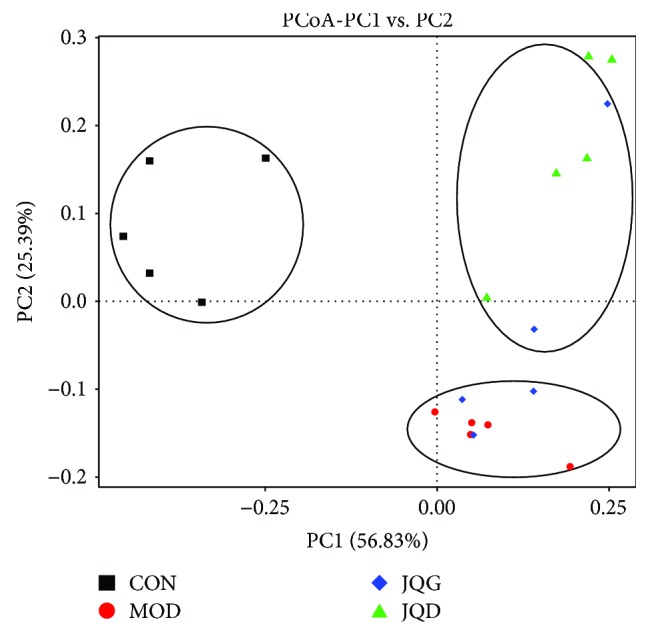
Bacterial communities were clustered using the principal coordinates analysis (PCoA). The first two principal coordinates (PC1 and PC2) from the PCoA of weighted UniFrac are plotted for each sample. The percentage variation in the plotted principal coordinates is indicated on the axes. Each spot represents one sample, and each group of mice is denoted by a different colour.

Since the composition of the gut microbiota is associated with T2DM [20], we determined the effect of JQJT tablets on the composition of the gut microbiota using the 16S rRNA gene sequencing analysis on an Illumina HiSeq platform. A total of 1,163,840 raw reads were obtained from 20 fecal samples. After performing quality control procedures to remove low-quality sequences, 1,148,860 reads were delineated into 2130 operational taxonomic units (OTUs) subjected to further analysis. The overall composition of the bacterial community in the different groups was clustered using PCoA (Figure 2), which was gained from the sequences at an OUT level of 97%. We demonstrated that JQJT tablets could regulate the gut microbiota in type 2 diabetes mice.

**Figure 3:**
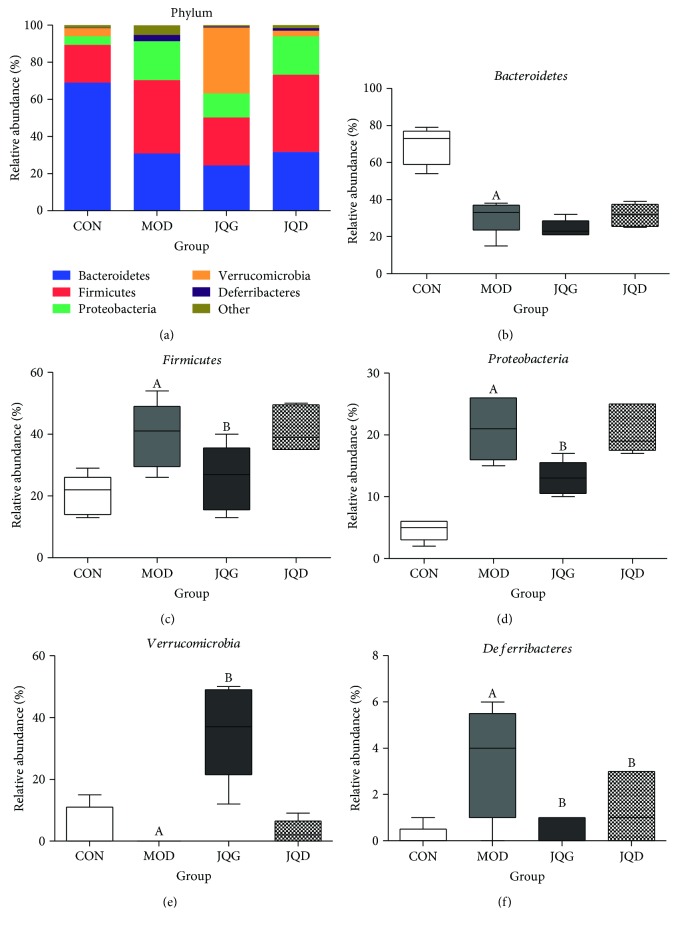
JQJT tablets modulated the gut microbiota in type 2 diabetes mice. Relative abundance distribution of operational taxonomic unit (OTU) sequences (98% level) at bacterial phylum levels (a). The relative abundance of *Bacteroidetes* (b), *Firmicutes* (c), *Proteobacteria* (d), *Verrucomicrobia* (e), and *Deferribacteres* (f). All data are expressed as mean ± SEM (*n* = 5 mice/group). Data with different superscript letters are significantly different (*P* < 0.05) according to post hoc one-way ANOVA.

In our study, the results were in accord with the research that demonstrated that the decreased diversity of the gut microbiota was connected with obesity and diabetes [21]. Our results showed that the number of bacterial species in diabetic mice was decreased. Interestingly, after the administration of JQJT tablets, the number of bacterial species was also decreased. So, next, we analyze the gut microbiota from the 5 selected bacterial phyla, which occupy 98% of the total gut bacteria, including *Bacteroidetes*, *Firmicutes*, *Proteobacteria*, *Verrucomicrobia*, and *Deferribacteres* (Figure 3(a)). We observed that in contrast with the CON group mice, the MOD group mice had a greater abundance of *Firmicutes* (*P* < 0.05), *Proteobacteria* (*P* < 0.05), and *Deferribacteres* (*P* < 0.05) and a lower abundance of *Bacteroidetes* (*P* < 0.05) and *Verrucomicrobia* (*P* < 0.05). After the administration of JQJT tablets, the abundance of *Verrucomicrobia* had a significant growth and the abundance of *Proteobacteria* (*P* < 0.05) and *Deferribacteres* (*P* < 0.05) declined in diabetic mice (Figures 3(b)–3(f)). This suggests that JQJT tablet treatment leads to a significant change in the gut microbiota of diabetic mice. So we considered that JQJT tablets alter the composition of the gut microbiota in parallel with its antidiabetic effect.

**Figure 4:**
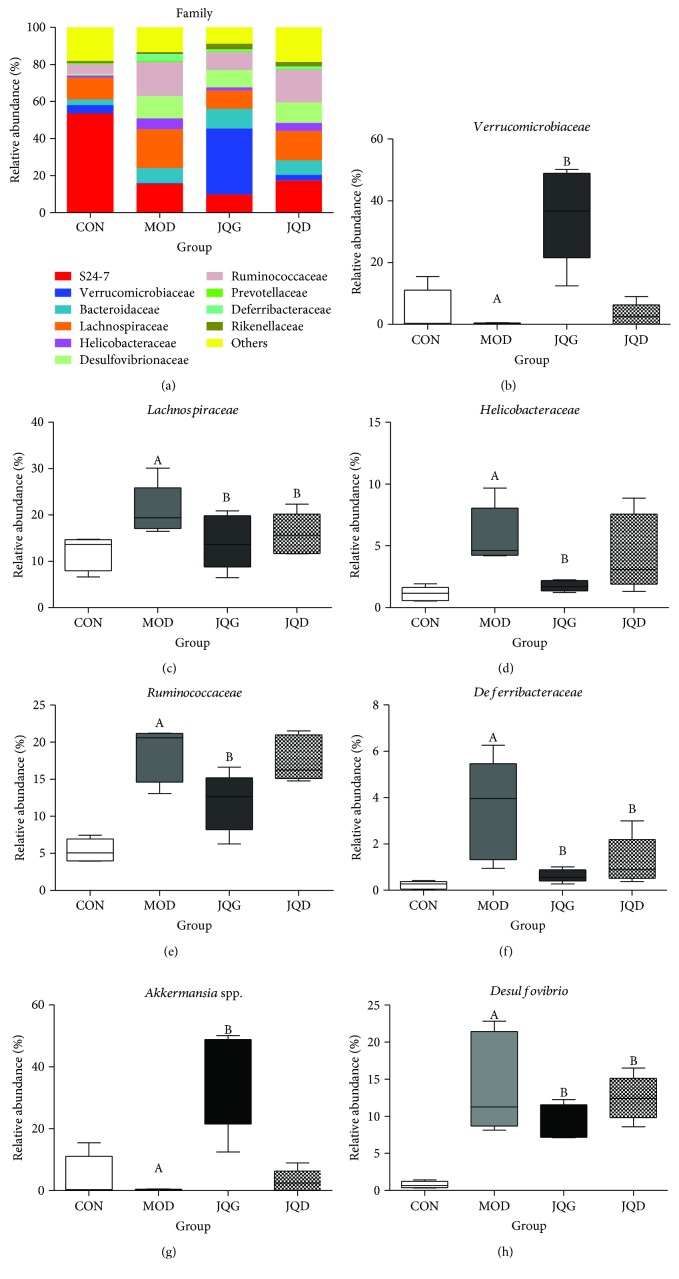
Taxonomy classification of reads at bacterial family levels in different groups (a). Only top 10 enriched class categories are shown in the figure. The relative abundance of *Verrucomicrobiaceae* (b), *Lachnospiraceae* (c), *Helicobacteraceae* (d), *Ruminococcaceae* (e), *Deferribacteraceae* (f), *Akkermansia* (g), and *Desulfovibrio* (h). All data are expressed as mean ± SEM (*n* = 5 mice/group). Data with different superscript letters are significantly different (*P* < 0.05) according to post hoc one-way ANOVA.

At the levels of bacterial families, totally, 185 families were identified from 20 mouse fecal samples; among them, the abundance of 39 OTUs was altered in the MOD group. After the administration of JQJT tablets, the abundance of 42 OTUs was enhanced or reduced. As shown in Figure 4(a), the top 10 families in mouse gut microbiota are *S24-7*, *Verrucomicrobiaceae*, *Bacteroidaceae*, *Lachnospiraceae*, *Helicobacteraceae*, *Desulfovibrionaceae*, *Ruminococcaceae*, *Prevotellaceae*, *Deferribacteraceae*, and *Rikenellaceae*. Among these families, the abundance of *Verrucomicrobiaceae* was lower in the MOD group than in the CON group. After JQJT tablet treatment, the abundance of *Verrucomicrobiaceae* was significantly increased with respect to the MOD group. The abundance of *Lachnospiraceae*, *Helicobacteraceae*, *Ruminococcaceae*, and *Deferribacteraceae* was reduced after JQJT tablet treatment with respect to the MOD group. It is noteworthy that there was no significant difference of the abundance of *Rikenellaceae*; however, this family was significantly increased after JQJT tablet treatment (Figures 4(b)–4(f)).

At the levels of bacterial genera, the abundance of *Akkermansia* spp. (*P* < 0.05) was significantly increased and the abundance of *Desulfovibrio* (*P* < 0.05) was significantly reduced after the administration JQJT tablet (Figures 4(g) and 4(h)). *Akkermansia* spp. are closely connected with inflammation [22], and the abundance was much lower in obesity and T2DM. And *Desulfovibrio* is related to the gut barrier [23]. We then analyzed the abundance of *Akkermansia* spp. and *Desulfovibrio* after the administration of JQJT tablets. The result showed that JQJT tablets rescued these changes in MOD mice, restoring the levels to those seen in model group mice, especially *Akkermansia* spp. and *Desulfovibrio*, which may play a key role in increasing insulin sensitivity and restoring the gut barrier. These results suggest the possibility that the antidiabetic effect of JQJT tablets may contribute to the modulation of gut microbiota.

### 3.3. JQJT Tablets Increase the Butyric Acid in Type 2 Diabetes Mice

**Figure 5:**
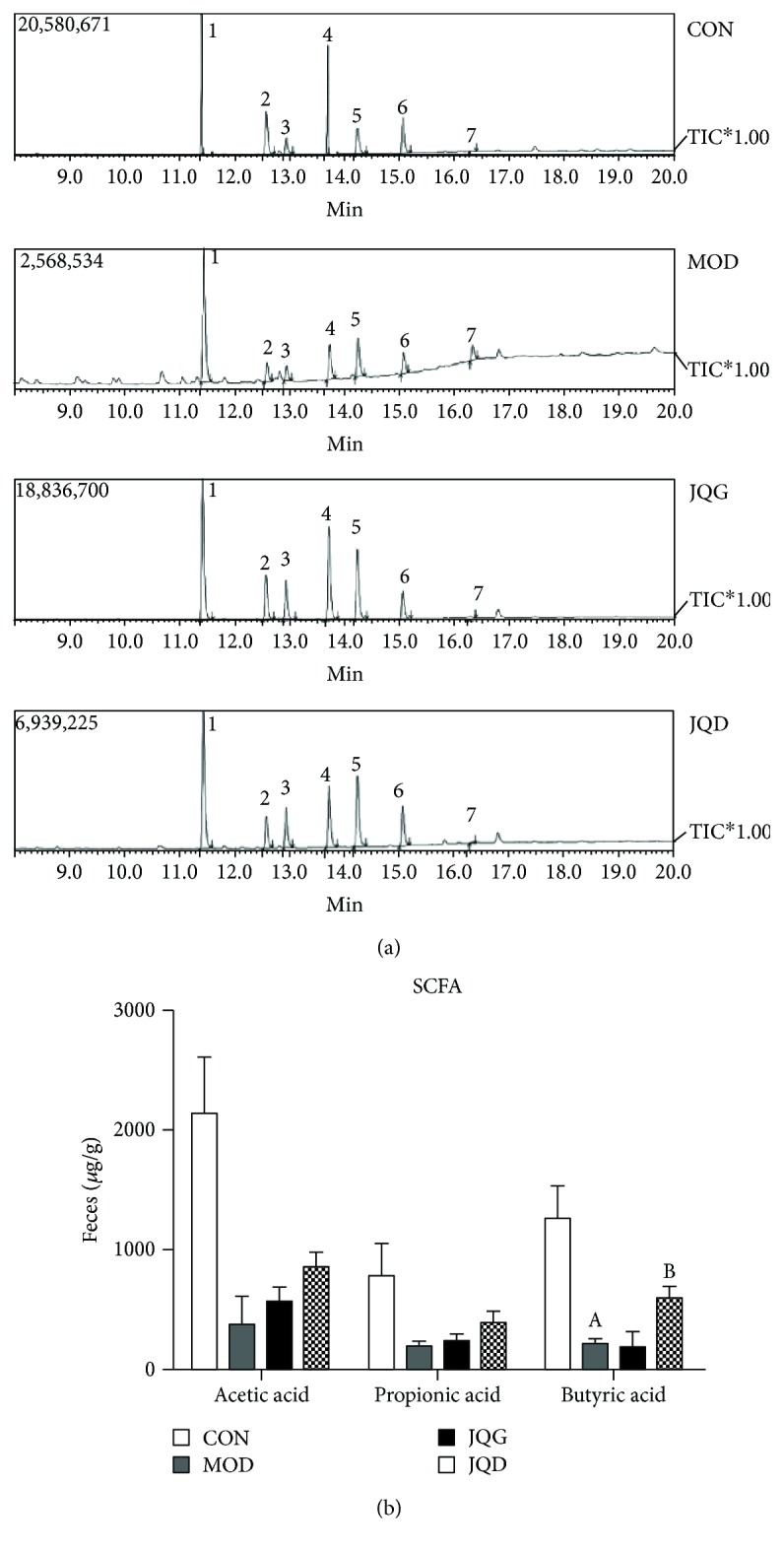
JQJT tablets increase the butyric acid in type 2 diabetes mice. Total ion chromatograms (TICs) of feces from different groups (a) and the main SCFA levels in feces quantified with different groups (b). All data are expressed as mean ± SEM (*n* = 5 mice/group). Data with different superscript letters are significantly different (*P* < 0.05) by one-way ANOVA.

Gut microbiota is associated with the production of SCFAs, which were beneficial for the improvement of gut barrier function and insulin resistance [24, 25]. So, we assessed the effect of JQJT tablets on SCFAs by HS-GC/MS. The result showed that seven components were identified in the SCFAs of mouse feces by matching their mass spectra with those of reference compounds recorded in NIST MS library and confirmed by reference substance, including acetic acid, propionic acid, butyric acid, isobutyric acid, pentanoic acid, isovaleric acid, and hexanoic acid. The total ion chromatograms (TICs) of SCFAs from 4 groups are shown in Figure 5(a). It could be observed that acetic acid, propionic acid, and butyric acid were the main components of SCFAs. So these components were calculated by a standard curve, and the result showed that acetic acid, propionic acid, and butyric acid were increased after the administration of JQJT tablets; in particular, butyric acid was significantly increased with respect to the MOD group (Figure 5(b)). It indicated that the modulation of gut microbiota by JQJT tablets can promote the production of SCFAs, especially butyric acid, which provided energy and nutrition for the intestinal epithelium. It is important for maintaining the integrity of the gut barrier.

### 3.4. JQJT Tablets Improve the Gut Barrier Function

**Figure 6:**
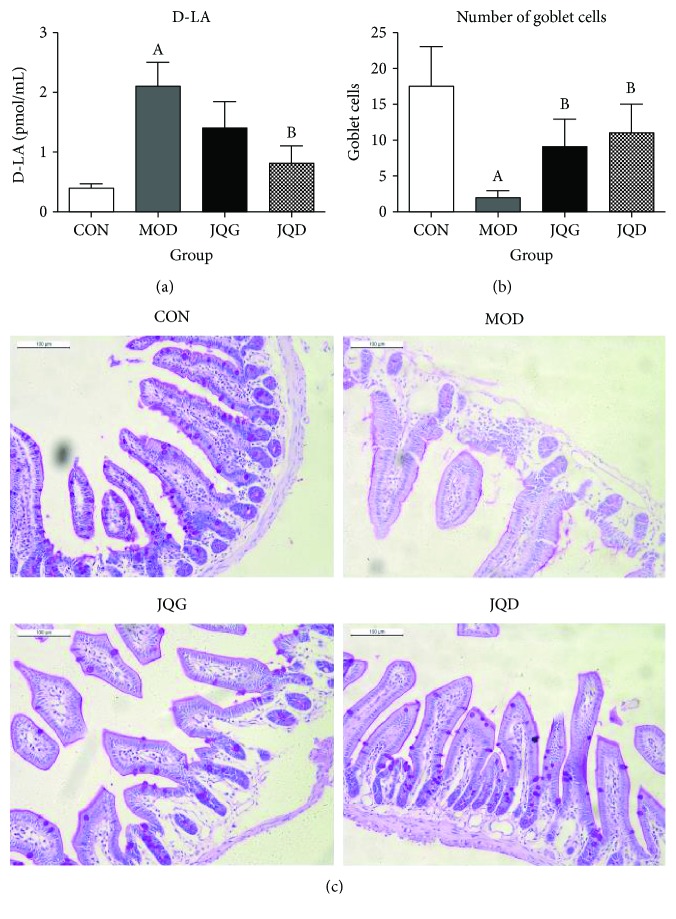
JQJT tablets improve the gut mucosal barrier function. Intestinal permeability was measured though the serum level of D-LA (a). JQJT tablet increases the number of goblet cells expressed as the number of goblet cells per villus (b). The goblet cells of the ileum were measured by histological analyses after PAS staining (c). All data are expressed as mean ± SEM (*n* = 5 mice/group). Data with different superscript letters are significantly different (*P* < 0.05) by one-way ANOVA.

Since there is a close association between SCFAs and the gut barrier function, next we evaluated the effect of JQJT tablets on gut barrier function by using D-lactic acid (D-LA) leakage experiment [26]. Serum samples were collected 4 h after the oral administration of D-LA. These samples were assayed for D-LA by using an enzymatic-spectrophotometric assay. The results showed that the level of D-LA was significantly increased in the model group mice. However, after 5-week treatment of JQJT tablets, the levels of D-LA were significantly decreased (Figure 6(a)). The result provided primary evidence that JQJT could improve the gut barrier function.

To provide further evidence that JQJT tablets improved the gut barrier function, PAS staining was used to check the number of goblet cells. Our results showed that JQJT tablet treatment increased the number of PAS-positive goblet cells (Figure 6(c)). We also counted the number of PAS-positive goblet cells per villus. An average of 17.5 ± 5.4 goblet cells was seen in control mice, which was higher than that in model group mice (2.1 ± 0.9) (Figure 6(b)). Interestingly, JQJT tablet treatment significantly increased the number of goblet cells (JQG 11.2 ± 3.8 and JQD 9.1 ± 3.5 vs. model 2.1 ± 0.0; *P* < 0.05), suggesting that JQJT tablets increased the goblet cell population. Taken together, these results indicated that JQJT tablet treatment could improve the gut barrier function.

### 3.5. JQJT Tablets Suppress the Inflammation in Type 2 Diabetes Mice

**Figure 7:**
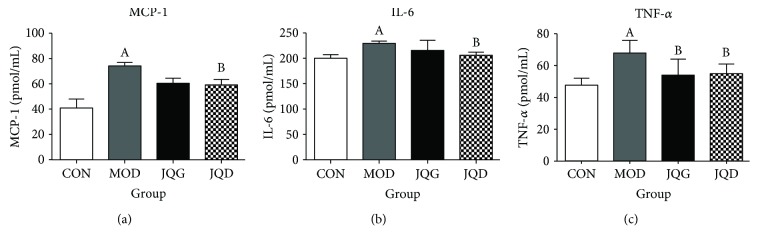
JQJT tablets reduce inflammation in type 2 diabetes mice. After the administration of JQJT tablets, the inflammatory factors MCP-1 (a), IL-6 (b), and TNF-*α* (c) were determined by ELISA. All data are expressed as mean ± SEM (*n* = 5 mice/group). Data with different superscript letters are significantly different (*P* < 0.05) by one-way ANOVA.

Low-grade inflammation is the most important characteristics of T2DM and closely related to the occurrence of insulin resistance. And impairments in gut barrier function can cause systemic inflammation. Therefore, we investigated the effects of JQJT tablets on the inhibition of inflammatory response by checking the levels of TNF-*α*, IL-6, and MCP-1 in serum by ELISA kits. Our results showed that the levels of TNF-*α*, IL-6, and MCP-1 were significantly upregulated in the model group compared with the control group. The administration of JQJT tablets clearly decreased the secretion of TNF-*α*, IL-6, and MCP-1 (Figures 7(a)–7(c)). These results illustrated that JQJT tablets could suppress the inflammation in type 2 diabetes mice.

## 4. Discussion

We demonstrated a role for JQJT tablets in modulating the composition of the gut microbiota, promoting the production of SCFAs, improving the gut mucosal barrier function, and alleviating the inflammation in diabetic C57BL/6 mice in the present study, and these may involve the action of JQJT tablets on the improvement of insulin resistance. Based on these results, we firstly provided some evidence that the insulin resistance improvement effect of JQJT tablets is more likely regulated by gut microbiota.

In the present study, HFD/STZ-induced type 2 diabetes model mice were employed. Consistent with a previous study [10], we first demonstrated that JQJT tablet treatment significantly improved the insulin resistance. However, the low bioavailability of the chemical composition in JQJT tablets makes it hard to explain the pharmacological mechanisms.

Accumulating studies indicated that gut microbiota regulation could improve the insulin resistance in type 2 diabetes mellitus. In particular, oral medications could regulate the gut microbiota directly, and the effects of JQJT tablets on gut microbiota regulation were evaluated by 16S rRNA gene sequencing. The study showed that *Akkermansia* spp. were remarkably increased while *Desulfovibrio* was significantly decreased after the administration of JQJT Tablets. *Akkermansia* spp. is mucin-degrading bacteria and has the ability to produce short-chain fatty acids (SCFAs) [27], which provide energy and nutrition to the intestinal epithelium, mitigate inflammation, and regulate glucose metabolism [28]. And reports also claimed that an appropriate amount of *Akkermansia* spp. inversely correlated with inflammation, altered adipose tissue metabolism, and metabolic disorders [29, 30], which played an important role in improving the insulin sensitivity [31]. *Desulfovibrio* is considered opportunistic pathogens and has been linked to some inflammatory diseases [32, 33]. *Desulfovibrio* is sulfate-reducing bacteria that can produce endotoxins and has the capacity to reduce sulfate to H_2_S [34]; H_2_S has been shown to disrupt energy metabolism in the gut epithelium [35], thereby damaging the gut barrier [36]. Our results showed that the model group detected little *Akkermansia* spp. and high richness of *Desulfovibrio*; however, the richness of *Akkermansia* spp. was significantly increased and *Desulfovibrio* was observably decreased after JQJT tablet treatment. Therefore, an appropriate abundance of *Akkermansia* spp. and a reduction of *Desulfovibrio* by JQJT tablets promote the production of SCFAs and protect the gut barrier function, which were good for improving the insulin resistance in T2DM.

Gut microbiota produces SCFAs through fermented dietary fiber. SCFAs were important for health, and deficiency in SCFA production is associated with type 2 diabetes mellitus [37]. Butyrate, in particular, is one of the preferred energy sources of the intestinal epithelium [38]. The increase in butyrate triggers intestinal gluconeogenesis, improving glucose and energy homeostasis and reducing hepatic glucose production, appetite, and body weight, meanwhile increasing epithelial barrier function. Modulation of the gut microbiota by JQJT tablets promotes the production of SCFAs, especially butyric acid, which provided energy and nutrition for the intestinal epithelium. It is important for maintaining the integrity and the function of the gut barrier. This result implies that JQJT tablets may improve the gut barrier function. Fortunately, the experiment about the gut barrier is in accord with our thoughts.

The intestinal barrier disruption plays a key role in the pathogenesis of diabetes, and the barrier function of the gut was important to the low-grade inflammation [39]. D-LA was the product of intestinal bacterial fermentation of lactic acid, which is rarely absorbed under normal circumstances, only when the intestinal mucosal integrity is damaged; the level of D-LA in serum could significantly increase. So the level of D-LA was checked after JQJT tablet treatment. Our results showed that the serum level of D-LA remarkably increased in the model group, which reflected an increased intestinal permeability in type 2 diabetes. JQJT tablet treatment significantly decreased the serum level of D-LA, which indicated that JQJT tablets could promote the integrity of the intestinal mucosa. On the other hand, goblet cells could secrete mucus and are one of the important selective barriers of gut barrier function [40]; we then checked the goblet cells in each group by PAS staining, and our results showed that the number of PAS-positive goblet cells was increased after JQJT tablet treatment. Based on these results, we suggest that the gut mucosal barrier function might be a contributing factor to the inflammation-inhibiting effect of JQJT tablets.

After the identification of the improving effect of JQJT tablets on the gut barrier, since insulin resistance was closely related to the low level inflammatory response in type 2 diabetes we evaluated the effect of JQJT tablets on inflammation by checking the levels of IL-6, TNF-*α*, and MCP-1. Our results revealed that the levels of TNF-*α*, IL-6, and MCP-1 were observably decreased after the administration of JQJT tablets, which indicated that JQJT tablets could inhibit the inflammatory reaction in type 2 diabetes. These results demonstrated that JQJT tablet treatment improves insulin resistance which was related to the inhibition of low-grade inflammation.

In our study, both the low and high doses of JQJT tablets have an effect on gut microbiota, alleviating inflammation and insulin resistance, but the dosage dependence is not significant. Gut microbiota is a large and complicated ecosystem, and traditional Chinese medicine has the characteristics of multiple pathways and multiple targets. Therefore, combined with the above two points, we speculated that a high dose and a low dose may affect insulin resistance in type 2 diabetic mice through different ways. A low-dose treatment modulates gut microbiota to produce butyric acid, improving the insulin resistance. Butyric acid repairs the damaged intestinal mucosal cells and restores the intestinal mucosal integrity. At the same time, a low-dose treatment can increase intestinal goblet cells to produce more mucus to form a mucus layer, reduced the intestinal permeability, and prevented the harmful substances from entering into the body that caused the inflammatory response, thereby inhibiting low-grade inflammation, while the high-dose treatment changed the gut microbiota differently from the low-dose treatment. Maybe the high dose treatment of JQJT tablets improves the insulin resistance through other pathways. That is what we will study for the future experiment.

## 5. Conclusions

Our present study firstly provided the evidence that JQJT tablets, a clinically commonly used Chinese patent medicine, could improve T2DM insulin resistance by regulating the gut microbiota, promoting the production of SCFAs, and the mechanism was related to increasing gut barrier function subsequently reducing the host inflammatory reaction.

## Data Availability

The data used to support the findings of this study are available from the corresponding author upon request.
